# Using Social Signals to Predict Shoplifting: A Transparent Approach to a Sensitive Activity Analysis Problem

**DOI:** 10.3390/s21206812

**Published:** 2021-10-13

**Authors:** Shane Reid, Sonya Coleman, Philip Vance, Dermot Kerr, Siobhan O’Neill

**Affiliations:** School of Computing, Engineering and Intelligent Systems, Ulster University, Derry/Londonderry BT48 7JL, UK; sa.coleman@ulster.ac.uk (S.C.); p.vance@ulster.ac.uk (P.V.); d.kerr@ulster.ac.uk (D.K.); sm.oneill@ulster.ac.uk (S.O.)

**Keywords:** human behaviour analysis, social signal processing, video processing, bias detection, ethical AI, machine learning

## Abstract

Retail shoplifting is one of the most prevalent forms of theft and has accounted for over one billion GBP in losses for UK retailers in 2018. An automated approach to detecting behaviours associated with shoplifting using surveillance footage could help reduce these losses. Until recently, most state-of-the-art vision-based approaches to this problem have relied heavily on the use of black box deep learning models. While these models have been shown to achieve very high accuracy, this lack of understanding on how decisions are made raises concerns about potential bias in the models. This limits the ability of retailers to implement these solutions, as several high-profile legal cases have recently ruled that evidence taken from these black box methods is inadmissible in court. There is an urgent need to develop models which can achieve high accuracy while providing the necessary transparency. One way to alleviate this problem is through the use of social signal processing to add a layer of understanding in the development of transparent models for this task. To this end, we present a social signal processing model for the problem of shoplifting prediction which has been trained and validated using a novel dataset of manually annotated shoplifting videos. The resulting model provides a high degree of understanding and achieves accuracy comparable with current state of the art black box methods.

## 1. Introduction

It is estimated by the British Retail Consortium (BRC) that shoplifting has cost UK business over 900 million GBP in 2018. To combat this, retailers are being forced to spend increasing amounts of time and money on security measures, such as hiring security staff and using security tags on more expensive items. According to the British Retail Consortium, retailers in the UK have spent over one billion GBP on crime prevention in 2018, almost four times the amount spent in 2014. Yet despite this, theft is on the rise [1].

There are a number of prevention strategies that have been shown to be effective at deterring potential criminals, such as placing additional security guards at entrances and exits, placing desirable items behind a counter and ensuring that staff have clear lines of sight [2]. The installation of CCTV cameras is one commonly used security method which is often employed by retailers in order to deter criminals. However, research has shown that unless actively monitored, surveillance cameras prove ineffective at preventing crime [3]. Furthermore, the research conducted by Gill [4] showed that thieves use several techniques to avoid detection. These techniques included using their body to conceal theft, becoming immersed within a crowd or wearing a disguise such as a cap or a hoodie. Often professional thieves will walk through a store to find blind spots where they can conceal an item without being seen by surveillance cameras. It can be difficult for those monitoring the live footage to spot these techniques and prevent theft. This is then compounded by the fact that those monitoring the footage will quickly become fatigued and may miss important indicators if they have to monitor several cameras for prolonged periods [5].

The development of an automated system which can detect individuals who exhibit the behaviours associated with shoplifting and draw them to the attention of security staff could help to alleviate some of these challenges. The work conducted by Mascorro et al. [6] aimed to detect suspicious behaviour using a 3D CNN model, proposing a new method for processing surveillance footage by segmenting each video into three distinct sections:

- Strict crime movement—the segment of the video where the individual commits the crime.
- Comprehensive crime movement—the precise moment when an ordinary person can detect the suspect’s intentions.
- Pre-crime behaviour—the individual’s behaviour from the time they enter the store until the comprehensive crime movement begins.

**Figure 1:**
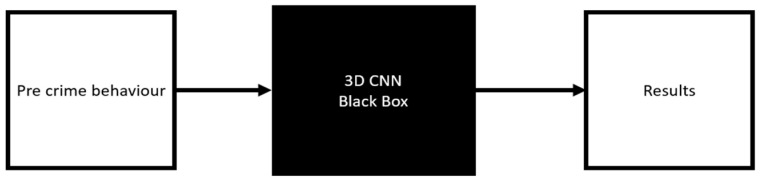
The black box model for shoplifting detection, where a raw video sequence is used to train a black box algorithm (such as a 3D CNN as in [6]) to detect suspicious individuals. The nature of these models makes them difficult to interpret and susceptible to bias in the training data.

They then trained their computer vision model to detect shoplifters using video footage taken from the pre-crime behaviour. Their proposed model achieves an accuracy of 75.7%. These results are encouraging as they demonstrate that an individual’s behaviour can be a strong indicator for predicting whether they are a potential criminal. However, while deep learning models [6,7], have been shown to achieve state of the art results for many computer vision problems, such models function effectively as a black box, as shown in Figure 1, making it almost impossible to determine how the algorithm came to its decision [8].

For high stakes problems, such as shoplifting detection, this lack of transparency is a major problem as it very difficult to determine whether the algorithm is learning to identify shoplifters based on their pre-crime behaviours, or if it is learning to identify shoplifters based on biases within the dataset [9]. The Committee of Experts on Internet Intermediaries (MSI-NET) at the Council of Europe has already outlined concerns around the admissibility of black box algorithms in criminal justice, and there are ongoing questions about potential human rights violations when using evidence from these systems in a court of law [10]. Rudin [11] clearly outlines the need for transparent, interpretable machine learning approaches for high stakes learning problems such as this.

**Figure 2:**
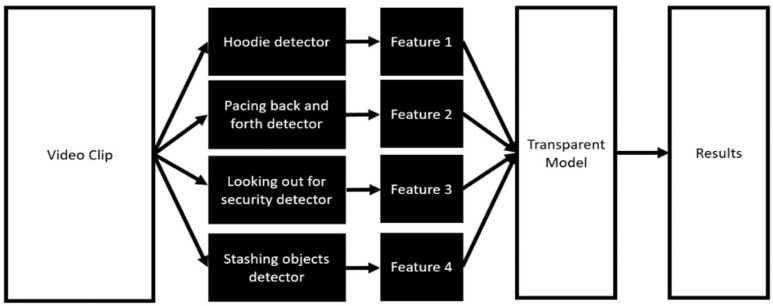
The transparent social signal processing model for shoplifting detection.

Psychology and criminology literature has shown that individuals who are likely to shoplift exhibit a number of easily observable behaviours, known as social signals [12]. Building an ensemble model based on the detection of these easily explainable features may provide the transparency needed to allow evidence from these models to be admissible in a court of law. Furthermore, we propose that by using a set of well-structured and meaningful features that correspond to social signals (as shown in Figure 2), we can determine which features are most useful for classification, enabling the development of more accurate and robust models.

The remainder of this paper will be structured as follows: in Section 2 we will discuss each of the key social signal modalities and how they relate to the problem of shoplifting detection. In Section 3 we will outline a set of 16 social signals that we propose may be used to determine potential shoplifters. We also outline a novel dataset of social signals that we have created by manually annotating videos taken from a dataset of real life shoplifting examples with the 16 social signals and perform some statistical analysis on this dataset. In Section 4 we will investigate how well these attributes perform as input features for a number of machine learning models and evaluate the performance using each of the social signal modalities. Finally, Section 5 will conclude the paper.

## 2. Social Signal Categories

According to Burgoon et al. [12], social signals can be grouped into five main categories: space and environment, face and eye behaviour, posture and gestures, vocal features and physical appearance. Any model may incorporate a combination of social signals from a number of these categories, each of which are described below.

### 2.1. Physical Appearance

Physical appearance is concerned with physical characteristics such as height and weight, as well as non-biometric features such as clothing and hair style. These characteristics can have an influence on how people are perceived by others. For instance, it is well documented in studies, such as that of Matsangidou and Otterbacher [13], that those who are perceived to be more attractive are assumed to possess more desirable personality traits and tend to be more successful occupationally. One very early study conducted by Steffensmeier et al. [14] found that individuals who were dressed in “hippy”-style clothing were significantly more likely to be reported by other customers for shoplifting than those who were wearing “straight” clothing such as a suit and tie. One possible ramification of such bias is that those who are monitoring surveillance footage may be more likely to focus on an individual based on their appearance rather than their behaviours.

However, in certain cases an individual’s appearance does warrant further investigation. For example, a shoplifter may wear a hat or a hood to conceal their identity on CCTV. Others may wear large baggy coats to easily conceal stolen items [4]. By using a social signal processing approach, we can detect the physical attributes that indicate that someone is a potential shoplifter.

### 2.2. Face and Eye Behaviour

This category is concerned with how individuals use facial expressions and facial gestures. In the most basic example, we smile when we are happy and frown when we are sad. The study conducted by Lasky et al. [15] investigated the ways in which shoplifters attempt to blend in with regular shoppers by masking their facial and eye gestures. In this study, they set up a simulated shoplifting scenario with shoplifters who were each fitted with an eye-tracking camera. Afterwards the participants were interviewed about their thoughts during the scenario. They found that shoplifters will often concern themselves with displaying socially ascribed facial expressions while inspecting the item they are planning to steal.

Unfortunately, the use of eye-tracking cameras is not feasible in a shop; however, there are techniques for head pose estimation which could be explored as an alternative for detecting these types of social signals from surveillance cameras [16].

### 2.3. Body Posture, Gestures and Actions

This category is concerned with how an individual’s body posture, communicative gestures and actions can help us to predict their future actions or behaviours. These sorts of attributes are unconsciously regulated and thus can be considered one of the most reliable indicators of an individual’s true intentions [12].

Shoplifters will often use different techniques in order to try to evade detection from security cameras. These can include going to surveillance blind spots and using sleight of hand techniques. Furthermore, the work of Lasky et al. [15] found that individuals may interact with shop staff before they attempt to steal an item in order to not arouse suspicion, and the work of Cardone and Hayes [2] found that shoplifters will often avoid staff after concealing an item in order to prevent being caught. One method that they will often use to do this is to pretend to be on a phone call as they exit the store [15].

**Figure 3:**
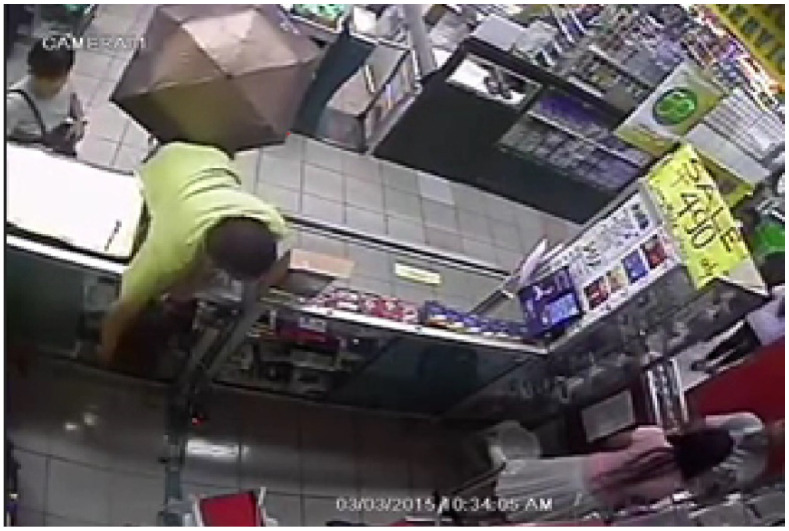
Sample frame from the UCF crimes shoplifting video dataset.

The work of Finklea [17] found that in cases of organized retail crime, often individuals will work in a group, with one or more individuals acting as a lookout for security staff surveillance cameras and other customers. Furthermore, these accomplices will often create diversions or distract store employees. A clear example can be found in the video frame in Figure 3, taken from the UCF crimes dataset [18]. In this real-life example, there are four individuals involved with the shoplifting attempt. One individual on the right is talking to and distracting the shop assistant; a second individual is standing in front with an umbrella raised to hide the attempt from other shoppers. A third individual stands to the left with an open bag ready to conceal the items. This helps to demonstrate the lengths that organized criminals will go to avoid detection.

### 2.4. Vocal Gestures

This category relates to both the lexical and prosodic features of an individual’s speech during an interaction (i.e., what they say and how they say it). Volume and rhythm are vocal features which are often used by a speaker for additional support in the communication/exchange of ideas within a social setting. Unfortunately, UK data protection laws make it much more difficult legally to collect and store audio data in a surveillance context [19]. Therefore, most retail surveillance cameras in the UK do not collect audio data. Therefore, the use of vocal features for the problem of shoplifting detection will be of limited practical value.

### 2.5. Space and Environment

This is the category concerned with the space that individuals inhabit and their use of interpersonal distance. The concept of socially distancing, while now familiar to almost everyone, has been studied extensively since at least the 1960s. In one of the seminal works in this area, anthropologist Edward T. Hall coined the term proxemics to describe the study of human space. Hall developed one of the first models for measuring proxemics in terms of eight separate measurements, such as physical distance, body orientation, the amount of touching, etc. [20]. Like postures and gestures, interpersonal distance is often unconsciously regulated and is considered a reliable method for determining the relationships between people.

Cardone and Hayes [2] studied how shoplifters perceive store environments, and outlined a number of techniques that can be used to help reduce the opportunities for shoplifting within a store. These include “hardening targets”, where expensive items are secured either behind a counter or using safes, cables or other reinforced materials; “natural surveillance”, where the store is designed in a way that enables store staff to have clear lines of sight throughout the store, e.g., by placing shelves perpendicular to the tills rather than parallel; “offender deflection”, where potential thieves are dissuaded by the presence of security officers or an established police presence; and “reducing frustration”, by preventing long queues and poor customer service.

From a shoplifting perspective this is an important category. Shoplifters will often attempt to avoid detection by moving away from store staff and other customers, by orienting themselves away from potential onlookers, and by concealing objects when they are in surveillance blind spots [15]. This avoidance behaviour may be a strong indicator that an individual is a potential shoplifter. Further, exhibiting non-avoidance behaviours may be an indicator that an individual is not a potential shoplifter [4].

## 3. List of Potential Social Signals for Shoplifting

In order to evaluate the effectiveness of social signals for the problem of shoplifting detection, and to evaluate the usefulness of each of the modalities, we have developed a set of 16 social signal attributes based on the current literature. We have then categorised these social signals into four of the modalities listed above using the following definitions:

1. Physical appearance—requires detection and classification from a single image of an individual.
2. Space and environment—requires detection and classification of multiple objects or individuals.
3. Body gestures and actions—requires detection and pose extraction over multiple video frames.
4. Face and eye behaviour—requires fine-grain pose estimation over any number of video frames.

If a social signal attribute could span two or more of these categories, then the label of the more difficult category was used.

To evaluate the effectiveness of these social signal attributes, and to evaluate each modality for the problem of shoplifting prediction, it was necessary to develop a novel dataset for this task. To do this we obtained a dataset of social signals by manually annotating videos from the University of Central Florida Crimes Dataset [18].

This dataset contains video clips for a large number of different criminal behaviours, such as arson and assault, as well as control videos where no crime was committed, though for these experiments we were only interested on the videos relating to shoplifting. For each video, a human observer manually annotated whether or not they observed a particular social signal. For a control we used the videos from the UCF Crimes Dataset which were based in a retail setting and where the individual being observed made a legitimate purchase.

We previously explored this dataset and published our results in [21]; however, here we will be expanding our analysis based on the social signal categories outlined in order to determine the best direction for future automation of these social signals, as well as releasing the dataset for public use to enable further research.

Each of the 16 attributes extracted from the dataset are described below, alongside some analysis of the frequency of the observed attribute within the UCF.

### 3.1. Physical Appearance

#### 3.1.1. Gender of the Individual

Whilst this first attribute could potentially be biased, we felt that it was important to include as the gender of an individual may influence the importance of the other appearance-based features. Additionally, we can use this attribute to find potential bias within the UCF dataset.

As Table 1 shows, there was a significant bias towards male shoppers in the non-shoplifting component of the dataset. While this may not necessarily reflect reality, it is important to be conscious of this bias when exploring the rest of the dataset.

#### 3.1.2. Gender of the Accomplice (Majority of Group)

This attribute may also be susceptible to bias; however, as certain actions might be suspicious depending on the gender of the individuals, we felt that it was important to include. Additionally, we can use this attribute to find potential bias within the dataset.

As Table 2 shows, in the cases of both the shoplifting and non-shoplifting classes, the gender of the accomplice was more likely to be found to be female than male. However, this difference is relatively small, and may simply indicate that female shoppers are more likely to shop together when compared to male shoppers.

#### 3.1.3. Are They Wearing a Hood or Baseball Cap or Other Clothing to Hide Their Appearance?

It stands to reason that individuals who are planning to shoplift may be more likely to wear clothing that can hide their appearance, such as a baseball cap or a hoodie, particularly if they have cased the store and know that there are surveillance cameras in place [4].

As Table 3 shows, the non-shoplifting individuals were actually more likely to be wearing some form of appearance-hiding clothing. This may indicate that this is a poor indicator of potential shoplifting. Due to the fact that this attribute can be significantly influenced by the gender of the individual, we decided to also include the frequency for the male and female subjects respectively.

As we can see, the results indicate that the shoplifting males are more likely to wear an article of clothing to hide their appearance.

Interestingly, the results show that female non-shoplifters were significantly more likely to wear appearance concealing clothing when compared to female shoplifters. One possible explanation for this may be due to the fact that more of the videos in the non-shoplifting class were recorded in countries where women traditionally wear head scarfs for cultural and religious reasons. These types of cultural norms may impact the reliability of this type of indicator, and therefore it is important to ensure that any deployed model takes these types of local features into account in order to avoid bias.

#### 3.1.4. Are They Wearing Baggy Clothing?

It is well documented in the literature that shoplifters will often try to hide an object on their person before leaving the store. Therefore, if an individual is wearing baggy clothing, this may indicate that they are planning to stash items in their pockets or elsewhere on their person [4].

The frequency of this attribute is shown in Table 4 below. As we can see, there is a clear corelation between wearing baggy clothing and shoplifting when measured across all subjects. When we examine the results by gender, we see that the male subjects who were not shoplifting were significantly less likely to be wearing baggy clothing when compared to male shoplifters. For the female subjects, almost all of those in the shoplifting set were found to be wearing baggy clothing, compared to an even split between baggy and non-baggy clothing amongst the non-shoplifting subjects. This may indicate that female shoplifters will often stash items in their clothing or in a bag when compared to male shoplifters, and that female subjects are more likely to wear baggy clothing in general.

### 3.2. Space and Environment

#### 3.2.1. How Many Others Are with Them?

Organized retail crime is often carried out by groups of two or more individuals [17] in order to facilitate the use of evasion techniques. Figure 3 demonstrates an organized group of four individuals carrying out a store theft where one individual is distracting the store clerk, another is blocking the view of other customers using an umbrella and a third is holding a bag open ready to stash the stolen goods. Organized groups of shoplifters may employ one or several of these types of techniques to reduce the risk of detection. For the purpose of this research, we defined an accomplice as anyone who interacted with or was closely related with the individual who was being observed, and who was not a member of staff. For instance, if two individuals entered the store and paced around it together or were clearly talking or interacting together within the scene then we would class them as accomplices of the observed individual.

As Table 5 shows, shoplifters were significantly more likely to have at least one accomplice when compared to non-shoplifting individuals. As we can see, the mean number of accomplices for non-shoplifters was 0.31, whereas for shoplifting individuals this value was 0.79. This indicates that having an accomplice could be a potential indicator that an individual is a potential shoplifter. However, given the limited size of the dataset a more thorough analysis would need to be done to confirm this hypothesis.

#### 3.2.2. Are Staff Members Visible within the Video?

According to Cardone and Hayes [2], shoplifters are less likely to attempt to steal an item if there is a member of staff nearby. Furthermore, evidence has shown that employing security guards to stand near valuable items or moving high-value items closer to the registers reduces the number of incidences of theft for those items.

Table 6 shows the frequency at which we observed staff members being visible within a given scene. As we can see, in the majority of videos for both classes staff members were visible within the shot. However, individuals who were not shoplifting were much less likely to be in a scene with no staff members visible when compared to the shoplifting class. Whilst this attribute is somewhat dependant on where the camera is set up, this does give credence to the idea that shoplifters will attempt to find blind spots before taking an item. This may indicate that surveillance systems are more necessary to deploy to parts of a store where there are no staff members visible.

#### 3.2.3. What Is the Potential Difficulty of Stealing the Item?

One method which has been shown to reduce the incidences of store theft is to place high-value items closer to the tills or behind a counter [2]. Therefore, items in these locations may be more likely to be the target of organized criminals rather than impulsive shoplifters. For the purpose of our investigation, we classified the difficulty of stealing an item on a scale of one to three, where one meant the item had very little security and three meant the object had a high degree of security. This score was determined depending on whether the item was kept behind the counter, how far the item was from the entrance/exit to the store and how likely the item was to have a security tag.

Table 7 shows the frequency of these scores as observed in the UCF Crimes Dataset. Interestingly these results show that shoplifters were more likely to attempt to take higher-value items rather than lower-value items. This may indicate that shoplifters are more likely to attempt to implement a high-stakes, high-reward strategy when it comes to theft. The results for the non-shoplifting class indicate that the types of items were distributed somewhat normally for what we would expect in retail shopping, i.e., most purchases were of small items, with few purchases of medium- and high-value items.

### 3.3. Body Gestures and Actions

#### 3.3.1. Duration of Time Spent in the Video

Individuals who are contemplating shoplifting will often wait until the optimal moment before they attempt to take an item. As a result, they may spend more time within the store and my take longer to perform certain actions when compared to a normal customer [2].

For this attribute we have measured the times each subject spent in the video and grouped them into 30-s intervals. As we can see from Table 8, the majority of subjects were observed to spend less than 60 s within the store in the case of both shoplifting and non-shoplifting videos, and there appears to be no direct corelation between each class. However, we did observe that a large number of the videos in the shoplifting set start with the observed individual already within the shot and ended before the individual left the store. Therefore, these results may be more the result of the cropping of the videos in the UCF Crimes Dataset than an actual trend of shoplifters spending less time in a store.

#### 3.3.2. Do They Appear to Hide What They Are Doing?

If an individual is planning to steal an item, they will almost always attempt to hide what they are doing from store staff and other customers in order to avoid being caught. They may attempt to do this using their body, by facing away from staff. Alternatively, they may attempt to hide behind an object shelving unit or a clothing rack [22]. In Figure 3, the individual is hiding behind an open umbrella that is being held by their accomplice.

As we can see from Table 9, this resulting feature is one of the strongest we have found so far, observed in 28 of the shoplifting videos and only one of the non-shoplifting individuals. This means that this feature alone could be used to predict shoplifting with an accuracy of approximately 77%. Therefore, one of the most important methods for shoplifting prevention would be automating the detection of this feature.

#### 3.3.3. Do They Place an Item Out of View into a Bag or Else Give an Item to Their Accomplice?

After taking an item, shoplifters will often try to conceal the object in a bag or in their pocket. Legitimate shoppers will often bag items after payment too; however, if an individual walks out of the store without bagging an item that may be an indicator that they are a legitimate customer [2,4].

Table 10 shows the results for this attribute. Interestingly, this appears to be one of the strongest indicators for shoplifting as well. We observed that for a large amount of the non-shoplifting videos, the customer purchased something small and carried it out of the store without placing it into a bag. However, it is worth considering that the frequency of non-shoplifters placing an item into a bag may vary depending on the retail context. For instance, customers who are doing a large grocery shop may be more likely to place items into bags when compared to customers who are stopping at a gas station, where they may only grab some confectionary that does not need to be bagged. Interestingly, however, the vast majority of shoplifters did place an item out of view, into a bag or pocket in order to conceal the item. Furthermore, we observed that in the majority of cases where the individual did not conceal the item, their attempt to shoplift was more akin to theft, where the individual grabbed an item and ran out the door, generally with a member of staff in pursuit. Therefore, this attribute may be a very strong indicator of shoplifting in retail environments where customers will rarely bag an item.

#### 3.3.4. Do They Pick Up an Item and Appear to Be Interested in It?

As we discussed in Section 2, the work of Lasky et al. [15] found that shoplifters will often pretend to inspect items that they have no intention of purchasing in order to avoid suspicion whilst they are waiting for an opportunity to conceal the taken items. Therefore, if an individual appears to be picking up an item and is interested in it, this may be an indicator that they are a potential shoplifter.

The result for this attribute is shown in Table 11. As we can see, this attribute was observed in almost all of the shoplifting videos, when compared to about 50% of the non-shoplifting videos. Therefore, individuals who do not appear to pick up an item and be interested in it may be less likely to be a potential shoplifter.

#### 3.3.5. Does the Video Show Them Interacting with Staff before Leaving?

The work of Lasky et al. [15] also found that shoplifters will leave the store quickly after stashing an item in order to avoid interacting with staff. They may also employ some form of social gloss (e.g., pretending to be on a phone call) in order to reduce the likelihood that someone will confront them or think that this behaviour is unusual.

The results for this attribute are shown in Table 12. As we can see, the non-shoplifting class almost all interacted with a member of staff before leaving the store, compared to the shoplifting class, where only ~1/3 of the observed subjects interacted with staff. In the majority of cases, the non-shoplifting individual was paying for an item before leaving. However, there were some observed cases where a non-shoplifters entered the store but did not make a purchase. Nonetheless, the results show that shoplifters are less likely to interact with staff before leaving than non-shoplifters.

### 3.4. Face and Eye Behaviour

#### 3.4.1. Are They Watching Staff or Other Customers?

Individuals who are attempting to shoplift will naturally want to minimise the chance of being seen by shop staff or other customers. Therefore, if an individual appears to be continually watching staff or other customers, this may be an indicator that they are waiting for the individual to move before making a shoplifting attempt. For the sake of this analysis, we define an individual as watching staff or other customers if they exhibited two or more of the following behaviours:

- Do they clearly look around for other customers or staff before picking up an item?
- Do they pick up an item while looking towards a member of staff?
- Does their accomplice look out for staff or other customers while they are picking up an item?
- Do they frequently look towards shop staff?
- Do they appear to be interested in the shopkeeper’s interactions with other customers?

The results are displayed in Table 13. As we can see, however, this attribute is a strong indicator that an individual is a potential shoplifter, with the majority of shoplifting individuals being observed to exhibit this behaviour and only a majority of non-shoplifting individual observed to have not exhibited this behaviour.

#### 3.4.2. Do They Exhibit Avoidance Behaviours?

In a manner similar to Section 3.3.5, shoplifters will often attempt to avoid interacting with shop staff or other customers in order to reduce the likelihood of being caught [15]. Therefore, if an individual appears to be exhibiting avoidance behaviours, this may be an indicator that they are a potential shoplifter. For this analysis, in order to determine whether or not the individual was exhibiting avoidance behaviours, we used a weighted metric where one point was added for each of the following four behaviours which indicate avoidance:

- Do they deliberately go to an area of the shop where they are not visible to the shopkeeper or security staff and stop and stay there for more than 5 s?
- Do they pick up an item while the shopkeeper’s back is turned to them?
- Do they appear to wait until other customers move away from them before picking up an item?
- Do they pace back and forth to a specific location before picking up an item?

Additionally, a point was subtracted if any of the following behaviours which indicate non-avoidance were observed:

- Do they pick up an item while visible to the shopkeeper?
- Do they pick up an item while visible to other shoppers?

If the final score for the metric was found to be one or higher, then the individual was considered to have exhibited avoidance behaviour.

Again, this attribute was found through a combination of more direct attributes, and the results are displayed in Table 14. As we can see, however, this attribute is a strong indicator that an individual is a potential shoplifter, with three-quarters of shoplifting individuals being observed to exhibit this behaviour and only a single non-shoplifting individual observed to have exhibited this behaviour.

#### 3.4.3. Is the Shopkeeper Distracted While They Pick Up an Object?

Another potential indicator that an individual is attempting to shoplift is if they pick up an item whilst the shopkeeper is distracted. As discussed above shoplifters often wait for the perfect opportunity to take an item without being spotted, whereas a regular shopper will take an item regardless of whether the shop staff are looking or not.

The results for this final attribute can be found in Table 15. As we can see, the non-shoplifting individuals were significantly less likely to pick up an item while the shopkeeper was distracted, when compared to the shoplifting individuals who were observed to do this in 50% of all cases. This is again another good indicator that an individual is a potential shoplifter.

## 4. Evaluation for Social Signal Models for Shoplifting Prediction

### 4.1. Experimental Setup

We have also evaluated how useful these features are for building ML models to predict whether an individual is likely to shoplift or not. In this way we can evaluate the predictive power of each of the categories and determine the best approach for future research. To do this we generate a feature vector for each video by concatenating the values recorded for each attribute in a given modality. For the majority of these attributes this value was binary; for the for the time-based attribute H, we use the time in seconds normalized between 0 and 1, and for the categorical attributes G and E we use the categorical label. For the gender attributes A and B, we used one hot encoding to ensure the model did not infer any ordered relationship between these values. This also enables us to set both values for attribute B to 0 when there was no accomplice observed.

For each set of attributes we generated models using three different supervised learning techniques: a support vector machine (SVM), which works by calculating the n-dimensional hyperplane which best separates each of the classes by the largest possible margin [23]; K-nearest neighbours (KNN), which is based on the idea that the class of a given object will be the same as those of its k-nearest neighbouring objects in the feature space [24]; and an adaptive boosted model (AdaBoost), which is a model based on training a large number of decision tree models and aggregating them to form a strong model [25]. We chose these particular methods for two reasons. Firstly, it is well known that different algorithms tend to perform differently for certain problems; therefore, it is worth exploring a range of modelling techniques to inform future research. Secondly, these particular models are known to perform well with small datasets, which is important here as the dataset consists of less than 100 examples.

For each model, five fold cross validation was used to validate the results, and the parameters for each model were optimised using a hyperparameter grid search, as shown in Table 16 below. We measured the performance of each model in terms of four metrics: accuracy, precision, recall and F1 score. The accuracy can be defined as a measure of the number of correctly classified instances:

(1) $$\mathrm{Accuracy}=\frac{\mathrm{TP}+\mathrm{TN}}{\mathrm{TP}+\mathrm{TN}+\mathrm{FP}+\mathrm{FN}}$$

Precision is used as a measure of the number of predicted individuals who were actually shoplifting defined as:

(2) $$P=\frac{\mathrm{TP}}{\mathrm{TP}+\mathrm{FP}}$$

Recall can be defined as the number of shoplifters in the dataset that were correctly predicted by our model:

(3) $$R=\frac{\mathrm{TP}}{\mathrm{TP}+\mathrm{FN}}$$

and the F1 score can be defined as the harmonic mean of precision and recall defined as:

(4) $$F1=2\times \frac{P\times R}{P+R}$$

where TP is the number of true positives, TN is the number of true negatives, FP is the number of false positives and FN is the number of false negatives. We define the positive case as those where the individual was a shoplifter and the negative case as those where the individual was not a shoplifter.

### 4.2. Physical Appearance

Table 17 shows the results when using only the physical appearance attributes, as outlined above, to train the models. As we can see, these models achieved a classification accuracy of over 75% when trained using the KNN approach. This is promising given that these physical appearance features should be the least difficult to extract from surveillance cameras.

### 4.3. Space and Environment

Table 18 shows the results when we trained the three models using only the space and environment features listed above. As we can see, there was a significant improvement in accuracy, compared with Table 16, for all trained models. The KNN model performed the best, achieving an F1 macro of 0.830, closely followed by the AdaBoost model at 0.800. This shows that there is a significant improvement in the ability to detect potential shoplifters when using features requiring this more detailed understanding of a scene.

### 4.4. Body Gestures and Actions

Table 19 shows the results when we trained the three models using the body gesture and action attributes as listed above. As we can see, in this case the KNN model performed most strongly, with an F1 score of 0.903. This was closely followed by the SVC model and the AdaBoost model. These scores were significantly higher than when using either of the two feature sets above, which indicates that extracting features for body gestures and actions may be one of the most reliable methods for determining if an individual is a potential shoplifter.

### 4.5. Face and Eye

Table 20 shows the results when we trained the three models using the final set of attributes, those relating to face and eye behaviour. As we can see, the models performed slightly worse than when using the body gestures and actions features; however, they performed significantly better that the two sets above. We can see that the KNN model performed worse than the AdaBoost model for these attributes, compared to the other sets, where the KNN model performed significantly better.

### 4.6. Combined Results

For the final set of results, we trained three models using the cumulative features from each of the four sets in increasing difficulty of extraction. Thus, starting with physical appearance features, we added space and environment features, then the body gestures and actions features and finally we trained three models using all of the extracted features. This gave us a good indication of the increase in performance these models could achieve if we increased the complexity of our model. The results for these experiments are shown in Table 21. As we can see, the models achieved a significant increase in performance at each split, rising from F1 scores in the range of 0.708 to 0.750 for the physical appearance attributes to a range of 0.934 to 0.956 when we trained the model using all of the attributes.

Overall, the KNN model appeared to perform the best; however, there was only a small difference between the three approaches at each split. Given the high F1 score achieved when using all of these attributes, we feel that provided each of the components could extract these features with a high degree of accuracy, this model could be used to screen for potential shoplifters. Table 22 shows the results of an ablation study, where we removed different sets of attributes and evaluated the effect. The results for when face and eye behaviour was removed are not included here as these results are equivalent to row 3 in Table 21.

As we can see, removing the space and environment attributes resulted in only a slight decrease in accuracy when compared with using all attributes. Additionally, removing the physical appearance-based attributes also had only a minor impact on the classification performance. Interestingly, removing the body gestures and actions attributes resulted in a significant decrease in the performance when compared with removing any of the other sets of attributes. However, we can see that even in this case the classification performance was still quite strong.

Finally, in Table 23 we show the model performance when using each set of attributes on their own for classification. As we can see, using only the body gestures and actions attributes resulted in the highest performing models, followed closely by the face and eye behaviours. The space and environment attributes alone performed poorly; however, the accuracy was still above 80% for all three models trained using these features. Interestingly, the physical appearance-based attributes performed the worst, achieving the highest accuracy score of 75.40%.

### 4.7. Comparison with Deep Learning Methods

In Table 24, we compare our method with two recently published deep learning methods. However, while these methods also used the shoplifting videos from the UCF Crimes Dataset, the UCF dataset contains over 1000 control videos not all of which are based in a retail setting. As such there has been no agreed subset of control videos identified as a benchmark for the specific problem of shoplifting detection. Therefore, the datasets used as a control in the compared studies may be slightly different from each other and from the one used in this study. Secondly, these results were reported for models which were trained using the raw video footage as opposed to our models which were trained on manually extracted social signal features. Therefore, the performance of our model is not that of a fully automated approach to shoplifting prediction, but rather represents the optimal accuracy that could be achieved if these social signals features were extracted with accuracy on par with that of a human labeller.

However, these results do indicate that our social signals-based method significantly outperformed both of the compared deep learning methods for the problem of shoplifting prediction. This is significant as it shows that a social-signals-based approach has the potential to significantly outperform current deep learning methods. Furthermore, this shows the strength of our selected social signals for this problem.

## 5. Discussion and Conclusions

In this paper we have outlined the need for a transparent approach to sensitive problems such as shoplifting detection. We have then outlined the social signal processing approach and proposed it as a potential way to overcome these problems. We have developed a set of social signal attributes based primarily on the works of Finklea [17], Cardone and Hayes [2], Lasky, Jaques and Fisher [15,22] and Gill [4], who have all proposed social signals that may predict shoplifting. These attributes have been grouped into social signal categories, as proposed by [12]. We then developed a novel dataset of these social signal attributes based on real-world shoplifting videos.

We have analysed the results from this dataset and evaluated the performance of these attributes when using a number of machine learning models to predict shoplifting. Our results demonstrate that models trained using these features can achieve very high performance for this problem. This is significant as it demonstrates that the transparency achieved by extracting these specific social signals does not come at a cost to performance. Furthermore, these results support the findings of those studies listed above that were used to generate this list of social signals.

There were a number of limitations to this study. Firstly, while the dataset used for this study—the UCF Crimes Dataset—is the largest open-source video dataset available for the problem of shoplifting detection, this dataset is still quite small, consisting of only 50 shoplifting videos. We recommend that a significantly larger dataset of shoplifting videos be compiled in order to validate these results. Secondly, during our analysis of this dataset, we noted that a number of the shoplifting videos were cropped short and did not show the subject entering or leaving the store. Additionally, in a number of the videos, the individual was obscured by walls or an object such as a shelf or clothing rack for part of the duration of the video. This makes it impossible to detect if any of our social signal attributes were exhibited during these portions of the video. We therefore recommend that future datasets remain uncropped, and if possible, that footage from multiple cameras is provided in order to help detect these attributes. Finally, we propose that more than one human labeller be used in order to improve the integrity of the dataset. In future work, we will attempt to identify additional social signal attributes that can be used for shoplifting detection. Furthermore, we hypothesise that the types of social signals exhibited may vary depending on the specific type of retail setting, and we intend to investigate this. Finally, we intend to begin the process of automation of the detection of these attributes for shoplifting prediction.

## Figures and Tables

**Table 1:** Frequency of the gender of the individual within the dataset.

Gender	Non-Shoplifting	Shoplifting
Male	31	25
Female	14	23

**Table 2:** Frequency of the gender of the accomplice within the dataset.

Gender	Non-Shoplifting	Shoplifting
Male	2	11
Female	4	15

**Table 3:** Frequency of individuals wearing clothing that may hide their appearance.

	Are They Wearing Clothing to Hide Their Appearance?					
	Non-Shoplifting (Male)	Shoplifting (Male)	Non-Shoplifting (Female)	Shoplifting (Female)	Non-Shoplifting (Total)	Shoplifting (Total)
Yes	5	6	11	3	16	9
No	26	19	3	20	29	39

**Table 4:** Frequency of shoplifters wearing baggy clothing.

	Are They Wearing Baggy Clothing or Carrying a Bag That Could Potentially Conceal an Item?					
	Non-Shoplifting (Total)	Shoplifting (Total)	Non-Shoplifting (Male)	Shoplifting (Male)	Non-Shoplifting (Female)	Shoplifting (Female)
Yes	16	33	9	12	7	21
No	29	15	22	13	7	2

**Table 5:** Frequency of the number of accomplices.

No. of Accomplices	Non-Shoplifting	Shoplifting
0	37	22
1	3	19
2	4	3
3	1	3
4	0	1
Mean	0.31	0.79

**Table 6:** Frequency of staff members being visible within a given scene.

Are There Staff Visible within Shot	Non-Shoplifting	Shoplifting
Yes	43	32
No	2	16

**Table 7:** Difficulty of stealing item.

Potential Difficulty of Stealing Item	Non-Shoplifting	Shoplifting
1	32	6
2	3	15
3	10	27
Mean	1.51	2.44

**Table 8:** Times spent by the subject in the video in seconds.

Time Spent in Video (s)	Non-Shoplifting	Shoplifting
0–30	16	13
30–60	15	17
60–90	7	4
90–120	6	4
120–150	0	3
150–180	0	0
180–210	0	2
210–240	0	3
240+	1	2

**Table 9:** Frequency of the attribute “Do they exhibit avoidance behaviours?”.

Do They Appear to Hide What They Are Doing	Non-Shoplifting	Shoplifting
Yes	1	28
No	44	20

**Table 10:** Do they place the item out of view into a bag?

Do They Place an Item Out of View into a Bag	Non-Shoplifting	Shoplifting
Yes	9	39
No	36	9

**Table 11:** Frequency of picking up an item and appearing to be interested in it.

Do They Pick Up an Item and Appear to Be Interested in It?	Non-Shoplifting	Shoplifting
Yes	21	39
No	24	9

**Table 12:** Does the video show them interact with staff before leaving?

Does the Video Show Them Interacting with Staff before Leaving?	Non-Shoplifting	Shoplifting
Yes	40	15
No	5	33

**Table 13:** Are they watching staff or other customers?

Are They Watching the Staff or Other Customers?	Non-Shoplifting	Shoplifting
Yes	12	36
No	36	9

**Table 14:** Do they exhibit avoidance behaviours?

Do They Exhibit Avoidance Behaviours?	Non-Shoplifting	Shoplifting
Yes	1	36
No	44	12

**Table 15:** Is the shopkeeper distracted while they pick up an object?

Is the Shopkeeper Distracted While They Pick Up an Object?	Non-Shoplifting	Shoplifting
Yes	6	24
No	39	24

**Table 16:** Hyperparameters optimized for each model.

Hyperparameters Used for Grid Search	
Model	Parameters
SVM	C = 1, Gamma = 0.01, Kernel = Linear
KNN	Distance Metric = Manhattan, Neighbours = 4, Weights = Distance
Random Forest	Number of estimators = (10, 25, 50, 75, 100), ‘learning rate’ = (0.5, 1.0, 1.5)

**Table 17:** Results for classification models when trained using the physical appearance attributes.

Model	Accuracy	Precision	Recall	F1 Score
SVM	71.1%	72.8%	71.2%	0.708
KNN	75.4%	76.8%	75.3%	0.750
AdaBoost	72.3%	73.5%	72.1%	0.719

**Table 18:** Results for classification models when trained using the space and environment attributes.

Model	Accuracy	Precision	Recall	F1 Score
SVC	80.6%	84.3%	80.1%	0.799
KNN	83.9%	86.8%	83.4%	0.830
AdaBoost	80.6%	82.0%	80.3%	0.800

**Table 19:** Results for classification models when trained using body gesture and action attributes.

Model	Accuracy	Precision	Recall	F1
SVC	89.2%	89.9%	89.3%	0.891
KNN	90.4%	90.9%	90.4%	0.903
AdaBoost	88.2%	88.7%	88.3%	0.882

**Table 20:** Results for the classification models when trained using face and eye behaviour attributes.

	Accuracy	Precision	Recall	F1 Score
SVC	86.0%	87.1%	86.3%	0.860
KNN	85.8%	86.9%	85.7%	0.856
AdaBoost	87.0%	87.7%	87.1%	0.870

**Table 21:** Results for the combined sets of attributes.

Attributes	Model	Accuracy	Precision	Recall	F1 Score
Physical Appearance	SVC	71.0%	72.8%	71.2%	0.708
	KNN	75.4%	76.8%	75.3%	0.750
	AdaBoost	72.3%	73.5%	72.1%	0.719
Physical appearance + Space and Environment Attributes	SVC	83.9%	86.1%	83.4%	0.831
	KNN	81.8%	86.4%	81.2%	0.807
	AdaBoost	76.3%	78.4%	75.8%	0.755
Physical Appearance + Space and Environment Attributes + Body Gestures and Actions	SVC	91.3%	92.3%	91.2%	0.912
	KNN	94.7%	95.5%	94.4%	0.946
	AdaBoost	92.5%	93.2%	92.7%	0.925
All Attributes	SVC	94.6%	95.3%	94.6%	0.945
	KNN	95.6%	96.2%	95.7%	0.956
	AdaBoost	93.5%	94.1%	93.4%	0.934

**Table 22:** Results when each set is removed.

Attributes	Model	Accuracy	Precision	Recall	F1 Score
All Attributes—Physical Appearance	SVC	92.46%	93.16%	92.44%	92.41%
	KNN	95.73%	95.89%	95.78%	95.72%
	Adaboost	91.29%	91.62%	91.33%	91.26%
All Attributes—Space and Environment Attribute	SVC	93.45%	94.50%	93.56%	93.35%
	KNN	94.56%	95.50%	94.67%	94.47%
	Adaboost	91.35%	92.17%	91.22%	91.27%
All Attributes—Body Gestures and Actions	SVC	88.13%	88.71%	88.22%	88.08%
	KNN	90.29%	90.97%	90.22%	90.22%
	Adaboost	87.02%	88.73%	86.78%	86.67%

**Table 23:** Results for each set individually.

Attributes	Model	Accuracy	Precision	Recall	F1 Score
Physical appearance	SVC	71.00%	72.80%	71.20%	70.80%
	KNN	75.40%	76.80%	75.30%	75.00%
	AdaBoost	72.30%	73.50%	72.10%	71.90%
Space and Environment Attribute	SVC	80.64%	84.31%	80.11%	79.87%
	KNN	83.86%	86.82%	83.44%	83.01%
	Adaboost	80.64%	81.98%	80.33%	79.96%
Body Gestures and Actions	SVC	89.24%	89.86%	89.33%	89.18%
	KNN	90.35%	90.86%	90.44%	90.30%
	Adaboost	88.19%	88.71%	88.33%	88.16%
Face and Eye Behaviours	SVC	86.02%	87.10%	86.33%	85.97%
	KNN	85.85%	86.86%	85.67%	85.62%
	Adaboost	87.02%	87.66%	87.11%	86.98%

**Table 24:** Comparison with deep learning approaches.

Method	Accuracy
Social Signals (Our approach)	95.6%
Martinez et al. (3D-CNN) [6]	75.7%
Ansari et al. (LSTM) [7]	74.53%

## Data Availability

The authors intend to release the dataset used for these experiments publicly alongside the publication of this manuscript. Please contact the corresponding author to obtain access to this dataset.
